# Charles Knower

**Published:** 1878-11

**Authors:** 


*Charles Knower, D. D. S., M. D.*, died in St. Louis, Mo., October 14th, 1878. Dr. Knower was a graduate of the Baltimore College of Dental Surgery, of the Class of 1859.

After receiving his dental degree, Dr. Knower commenced practice in his native city, where he had been a student of Dr. Isaiah Forbes. He was a successful practitioner, and his memory is held in high estimation by all who knew him. While engaged in the practice of dentistry he studied medicine and received the Degree of Doctor of Medicine.

